# Active Optical Sensors for Tree Stem Detection and Classification in Nurseries

**DOI:** 10.3390/s140610783

**Published:** 2014-06-19

**Authors:** Miguel Garrido, Manuel Perez-Ruiz, Constantino Valero, Chris J. Gliever, Bradley D. Hanson, David C. Slaughter

**Affiliations:** 1 Laboratorio de Propiedades Físicas (LPF)-TAGRALIA, Technical University of Madrid, Madrid 28040, Spain; E-Mail: constantino.valero@upm.es; 2 Aerospace Engineering and Fluids Mechanics Department, University of Seville, Ctra. Sevilla-Utrera km 1, 41013 Seville, Spain; E-Mail: manuelperez@us.es; 3 Department of Plant Sciences and Biological and Agricultural Engineering, Sensor and Instrumentation Lab, University of California, Davis, One Shields Ave, Davis, CA 95616, USA; E-Mails: cjgliever@ucdavis.edu (C.J.G.); bhanson@ucdavis.edu (B.D.H.); dcslaughter@ucdavis.edu (D.C.S.)

**Keywords:** optical sensors, tree stem detection, state tree classification, LIDAR, light curtain transmission

## Abstract

Active optical sensing (LIDAR and light curtain transmission) devices mounted on a mobile platform can correctly detect, localize, and classify trees. To conduct an evaluation and comparison of the different sensors, an optical encoder wheel was used for vehicle odometry and provided a measurement of the linear displacement of the prototype vehicle along a row of tree seedlings as a reference for each recorded sensor measurement. The field trials were conducted in a juvenile tree nursery with one-year-old grafted almond trees at Sierra Gold Nurseries, Yuba City, CA, United States. Through these tests and subsequent data processing, each sensor was individually evaluated to characterize their reliability, as well as their advantages and disadvantages for the proposed task. Test results indicated that 95.7% and 99.48% of the trees were successfully detected with the LIDAR and light curtain sensors, respectively. LIDAR correctly classified, between alive or dead tree states at a 93.75% success rate compared to 94.16% for the light curtain sensor. These results can help system designers select the most reliable sensor for the accurate detection and localization of each tree in a nursery, which might allow labor-intensive tasks, such as weeding, to be automated without damaging crops.

## 1. Introduction

Juvenile trees are propagated in a tree nursery and grown to usable size before transfer to a permanent orchard site. Similar to other agricultural crops, nursery tree production is affected by temperature, drought, and economic pressures on the production practices associated with labor requirements and pest control needs. Most of the nursery operations remain highly labor intensive and utilize minimal automation of mechanized practices. Although some processes have been mechanized and automated, many others have not. According to estimates based on the “Resource book on horticulture nursery management”, by Yashwantrao Chavan Maharashtra Open University, manpower accounts for 70 percent of the production costs of a horticultural nursery [1].

In nearly all tree nurseries, seedlings are grafted in the spring, pruned and grown during the summer and fall, and excavated the following winter for bare-root sale. To efficiently market these trees, the nursery must have a precise count of the number and size distribution of each cultivar. A sampling method is used by many nurseries when conducting inventories and the total number of trees in the field is estimated by counting a selected number of rows. An error that occurs during counting might cause serious marketing problems if the number of estimated trees in the sample is not close to the actual number of trees available in the nursery. Some nurseries count each tree in the field, which results in a labor-intensive operation [2]. With this method, an evaluation of the feasibility of the automation of these nursery tasks compared to the efficiency of manual labor would be beneficial for determining if automation might lead to a lower cost for the nursery, which would significantly benefit the industry.

Specialty crop producers are beginning to experience significant progress in automating tasks that had previously been the exclusive domain of major crops, such as wheat, soy, and rice. Nearly 30 years ago, Maw *et al.* [3] developed a photoelectric transducer for counting seedlings in containerized nursery operations. For the development of this study, they used a photo-interrupt sensor with a near-infrared (NIR) emitter and a phototransistor detector mounted in a stationary comb through which the trays were conveyed. The results indicated were an accuracy of 98% and an imprecision of 3% with a speed of 40 plants per second [3]. Today, vehicles are capable of driving autonomously along rows of fruit and nursery trees while incorporating a variety of sensors that increase management efficiency [4].

Kranzler [5] developed an optoelectronic tree seedling counter for use in forestry nurseries with similar systems. The system was composed of a light-barrier with multiple NIR light-emitting diodes (LED) on one side of the seedlings for illumination and a linear array of photodiodes on the other side for detection. In this way, a tree was counted as long as all of the detectors were blocked. An optical encoder for measuring linear displacement was coupled to a small tractor wheel where the sensor system was mounted. The results showed a count error with pine seedlings ranging from 4% to 58% depending on the sensor settings and diameter measurement error with wooden dowels ranging from 2.5% to 40.6%. Problems caused by needle formation and irregular stem structure limited the field measurement of diameter to counts within size categories [5].

Delwiche and Vorhees [2] developed an optoelectronic system to count and size fruit and nut trees in commercial nurseries. For this purpose, an optical sensor was designed using a high-power infrared laser for illumination. Similar to the study of Kranzler, a rotary encoder was used and was coupled to one of the wheels of the cart for displacement measurements. The signal processing was based on the comparison of the recorded signal with the background threshold; when the background threshold was exceeded, it corresponded to a tree trunk entering the field of view. Calibration tests showed that the system could measure the trunk diameter to 1.9 mm (three times the standard error of prediction) with a sensor placed 15 to 23 cm from the tree line. Leaves, low-level suckers, and weeds were observed as causing inaccurate counting and sizing [2].

Lasers were used as an optical sensor in a study by Kise *et al.* [6], who developed a targeted spray system for cutworm control in grapes that hit only the targeted trunk or posts. The system consisted of a laser sensor system for target recognition and a single-nozzle sprayer system, all of which were built on a small electric utility vehicle platform equipped with automated speed control and steering. The results showed trunk detection greater than 96% at operational speeds of up to 1.1 m·s^−1^ [6].

Kang *et al.* [7] adapted the concepts used by Kise *et al.* [6] in their targeted sprayer system to a low-cost, commercially available spray trailer. The automated trailer-based sprayer system consisted of a scanning laser-based trunk detection system and multi-nozzle sprayer controller installed on a modified trailer sprayer with both sides equipped to spray grape trunks in adjacent vineyard rows. When the laser sensor completed one full scan, the raw data, including the distance and measurement angle, were obtained and conveyed to the trunk's labeling and filtering function, which extracted the trunk information from the raw data. The labeling function evaluated each point's connectivity to adjacent points based on a fixed distance and determined the presence of an object (trunk or post). The tests showed that the laser scanner-based target recognition system could detect trunks and posts at all of the tested travel speeds. However, the detected trunk radius decreased linearly with increasing speed [6].

Following the same line of work one year later, Kang *et al.* [8] developed a sucker detection system to trigger a targeted spray application for vine-specific sucker control in grape vineyards. The sucker detection system consisted of a laser scanner for vine detection and color camera for imaging the suckers. The results from field tests showed that the developed system could identify more than 97% of the suckers at three travel speeds, from 1.6 to 3.2 km·h^−1^. The average accuracy of the sucker dimension measurement ranged from 83% to 88%. The root mean square error (RMSE) of the relative position of suckers to the corresponding trunk varied from 13 to 29 mm [8].

Previously studies have not assessed different optical sensors under the same conditions to determine the state of the tree (alive or dead) [9]. Under this premise, the main goal of this article was to evaluate the two optical sensors (laser and photoelectric) most commonly used for such assessments under the same nursery test conditions.

The objective of this study was to evaluate under the same nursery test conditions two different optical sensors and their data processing software to select the most reliable sensor for the accurate detection, localization and classification (alive or dead) of each one-year-old tree in the nursery. The use of these sensors will enable automated tree counting and potentially other future tasks with the same efficiency as manual labor and at a lower cost to the nursery.

## 2. Materials and Methods

### 2.1. Optical Sensors and Configuration

The optical sensors evaluated were a LIDAR (Light Detection And Ranging) sensor for determining the distance from a laser emitter to the tree using a pulsed laser beam based on the time-of-fight (TOF) and a photoelectric transmission barrier using 4 pairs of optical light curtain transmitters and receivers to evaluate the interruption by the tree of the light curtain between the two devices. All of the sensors were installed on a prototype vehicle, and they are examined in detail below.

#### 2.1.1. Laser Sensor

The laser sensor was the model LMS 221, SICK AG, Waldkirch, Germany, and its main characteristics are summarized in Table 1.

**Figure 1.**
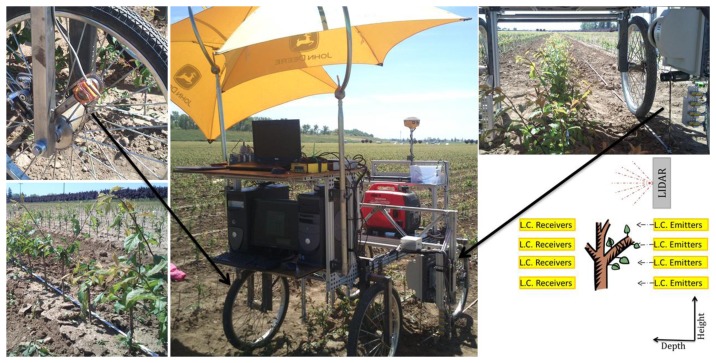
(**Middle**) Equipment mounted on the sensor platform that was used. (**Bottom left**) Tree stem detection. (**Top right**) Detail of the two sensors locations: Laser at the top and four light curtain emitters at the bottom. (**Bottom right**) Schematic of the LIDAR and light curtain orientation. (**Top left**) Detail of the encoder coupled by a timing chain to one of the wheels.

The laser was installed in a vertical orientation in the middle, right side of the prototype vehicle with a ground clearance of 56 cm and at a 50 cm distance from the centerline of the vehicle (Figure 1). The laser was positioned to scan the row of trees passing through the center of the vehicle. At this distance from the trees, and according with to the manufacturer's specification of the laser, the spot diameter of the laser beam was 3 cm with a distance between the individual measured points (spot spacing) of 1.8 cm.

#### 2.1.2. Light Curtain Sensor

The light curtain sensor was the model Mini-Beam SM 31 EL/RL, Banner Engineering Co., Minneapolis, MN, USA, and its main characteristics are summarized in Table 2.

Four light curtain emitters were installed vertically in the same line as the laser on the middle right side of the prototype vehicle with a height above ground of 12.7 cm to the lowest emitter and a 5.1 cm vertical spacing between each emitter. The four receivers were installed in the middle, left side of the prototype vehicle with a distance to the transmitter of 1.1 m so that the light curtain covered a vertical height from 12.7 cm to 28 cm above ground level (Figure 1).

The recording frequency of the light curtain was determined by the forward speed of the vehicle because the acquisitions of each pair of light curtain data (interruptions in the beam) were triggered by changes in the odometry encoder values (*i.e.*, by forward travel). A horizontal slotted aperture of 1.0 × 6.4 mm (AP31-040H) was installed in each light curtain sensor; use of these apertures allowed for a closer matching of the size and shape profile of the detected object (*i.e.*, trees).

#### 2.1.3. Sensor Acquisition Configuration

As previously mentioned, the sensors were installed on a manually powered prototype vehicle that was composed mainly of structural framing (to provide suitable robustness), four bicycle wheels, and a pair of horizontal shelves as support for the computers (one for each optical sensor). Placement of the sensors was along a vertical line, which eliminated the need to perform any offset calculations to compare the results between sensors (Figure 1).

A rotary encoder wheel was coupled by a timing chain to one of the wheels for vehicle odometry and used to conduct the evaluation and comparison of the different sensors (Figure 1). This arrangement provided a measurement of the linear displacement of the prototype vehicle along the row of tree seedlings and formed the spatial basis for each recorded sensor measurement.

To determine the most appropriate vehicle speed for the test, the lowest frequency and field of view of the two sensors were considered. With a LIDAR frequency of 10 Hz, and by scanning each tree stem (1 cm diameter) three times, it was concluded that the speed could be as high as 0.108 km/h (3 cm/s).

For the LIDAR acquisition system, the manufacturer's software was developed in C++, and it combined LIDAR, GNSS, and data received by the odometry encoder through an Arduino “UNO” device. For the light curtain acquisition system, the optical output signals were connected to a bidirectional digital module (NI 9403, National Instruments Co., Austin, Texas, USA), whereas the encoder signal was connected to a digital input module (NI 9411, National Instruments Co., Austin, Texas, USA). Both modules were embedded in an NI cRIO 9004 (National Instruments Co., Austin, Texas, USA), and all of the data were recorded using the Labview software program (National Instruments Co., Austin, Texas, USA).

**Figure 2.**
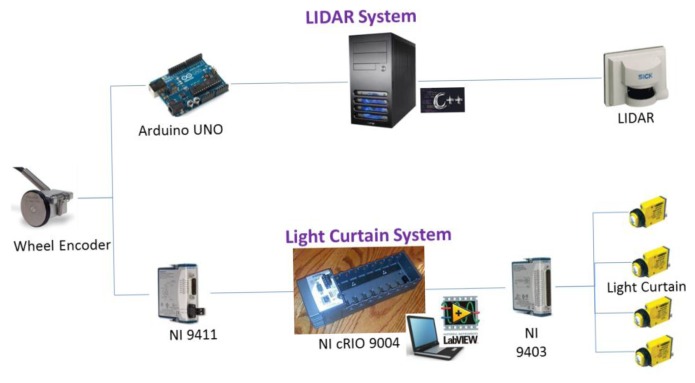
Devices and software used the two systems.

All of the necessary components for each of the aforementioned systems (LIDAR and light curtain, Figure 2) were recorded in a parallel fashion using the encoder value for future event synchronization and evaluation. In this way, two independent files were obtained from each optical sensor system and test.

### 2.2. Field Experiments

On 19 April, 2013, six field trials were conducted on one-year-old grafted almond trees at the nursery of Sierra Gold Nurseries in Yuba City, CA, United States. Each field test plot consisted of 20 m of data collected along the tree line with the use of the manually powered vehicle. Trees were located in beds that were 15 cm high, 61 cm wide, and 400 m long. The distance between trees was 20 cm, and the tree top height above the ground was approximately 22 cm. Because of the good ground conditions and their installation on the vehicle, the laser detections did not require correction by the IMU.

### 2.3. Method for LIDAR Tree Stem Characterization

After the field data had been recorded using the laser, processing was performed. An algorithm was written to convert the distances and angles from the LIDAR detection to 3D coordinates using the encoder value for the displacement of the vehicle.

**Figure 3.**
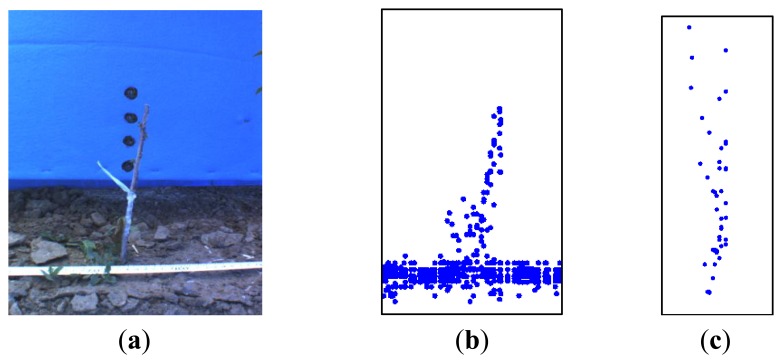
A sample of the LIDAR outlier that occurs at the boundary of an occlusion. (**a**) Image of the scanned tree. (**b**) Front view of the detection recorded of the tree by the LIDAR sensor. (**c**) Top view of the detection recorded of the tree by the LIDAR after ground removal.

The analysis of these initial values showed an outlier effect of the depth for laser values. According to previous studies: “An outlier is an observation that deviates so much from other observations as to arouse suspicion that it was generated by a different mechanism. Large errors or outliers can be caused by different sources and they are mainly measurements that do not belong to the local neighborhood and do not obey the local surface geometry. As the footprint of the laser beam is not a geometrical point, but an ellipse, when it hits a boundary of an occlusion (*i.e.*, the tree), it is divided into two parts, each of which radiate one of the front and the back surfaces incident to the occlusion boundary. Thus, the irradiance at this point would be a weighted average of the irradiance reflected by both surfaces” [10] and in the case of a tree row does not represent a true point on either the tree or the background, but is an artifact of the beam size and the size of the juvenile tree. The tree stem detection in our study showed this outlier effect (Figure 3) was enhanced because the object to be scanned (one-year-old grafted almond trees) was normally smaller than the spot diameter of the laser (according to the manufacturer's documentation, an LMS 221 had a spot diameter of approximately 3 cm at a 1 m distance) [11].

To reduce the error produced from the outlier detection's shape, the LIDAR tree stem detection task was performed in three steps: *data filtering* for delimiting the number of detections to the area of interest; a *calibration test I* for evaluating and selecting the tree stem identification parameters values included in the algorithm; and *validation* of the proposed methodology.

To extend the application range of this methodology in nurseries, it is necessary to consider the number of trees present in a field and their state as either alive or dead. For this purpose, tree classification was performed in two steps: *calibration test II and validation*. For this study, an off-line *Matlab* process was used with actual field data collected during the field tests. In its final version, the process will run on-line to adjust the mechanical weed implement without damaging the crop trees.

#### 2.3.1. Data Filtering

The 3D LIDAR data were filtered by removing unnecessary measurement data from the background, ground and all detections outside of the vehicle frame. To remove the unnecessary ground data, all of the detections with a height lower than 17 cm were removed. This height threshold was obtained by manual analysis of the data. To remove data from within the interest area (removal of background and measurements from outside the vehicle's frame), a boundary delimitation was performed for depth and height, and only the values with a LIDAR distance of 10 to 65 cm and with a height less than 10 cm above the LIDAR height (56 cm) were retained.

#### 2.3.2. Calibration Test I: Stem Identification and Selection of Parameter Values

Data provided by the 4 field experiment tests were used for the calibration test, which was composed of 373 trees. The methodology used for reducing the LIDAR outlier effect during tree stem detection was based on six different filter parameters (height threshold, encoder range, path increment, cut identification, jump, and blanking tree distance) and applied as follows:

1. Once the data were filtered, there was an initial height threshold applied to the remaining points (*height cut parameter*) to focus the study on the stems without considering leaves and branches, which strengthened the outlier shape effect.
2. The trees should be located where the number of detections is maximal, so a depth (perpendicular distance from the LIDAR) histogram evaluation was performed. Histograms were produced for every 10 encoder values and by selecting different range data (*encoder range parameter).*
3. The depth value obtained with the highest detection number for a histogram was related to the average value of the data encoder range. This defined a line, termed the tree line, which was then smoothed.
4. The tree path was obtained by adding and subtracting a defined value (path increment parameter) to this tree depth line.
5. A second threshold in height was applied (*cut identification parameter*) based on the detections at the starting point (after data filtering) that were inside the tree path.
6. A binary transformation was performed to determine the presence or absence of detections for each encoder value. These binary values were filtered by changing all of the absence values (that were between presence values) from absence to presence inside an evaluation window (*jump parameter*).
7. The initial, final and median encoder values were obtained from each presence series. The potential tree detection was removed when the distance in the encoder values from its midpoint to the previously detected tree midpoint was lower than a threshold (*blanking tree distance parameter*).
8. The median encoder value of each tree detected by the LIDAR sensor was compared with the actual values obtained manually during the tests. The real tree location and LIDAR tree detection were compared by proximity and deemed a success if the distance between them was less than 80 encoder values; otherwise it was considered to be a false positive (detected by the laser but not real) or negative (real tree not detected by the laser).

This methodology was conducted for the study of each parameter independently through an evaluation of the following parameters: height cut parameter from 18 to 25 cm, encoder range parameter from 127 to 5080 cm steps of 150 encoder values, path increment parameter, from 2.54 to 22.86 cm steps of 2 cm, cut identification parameter from 18 to 25 cm, jump parameter from 2.54 to 22.86 cm steps of 2 cm, and blanking tree distance parameter from 127 to 482.6 cm steps of 20 encoder values.

In each independent evaluation, the values of the other variables were set to their average value: 22 for height cut and cut identification, 1025 for encoder range, 5 for path increment and jump, and 125 for tree distance.

**Figure 4.**
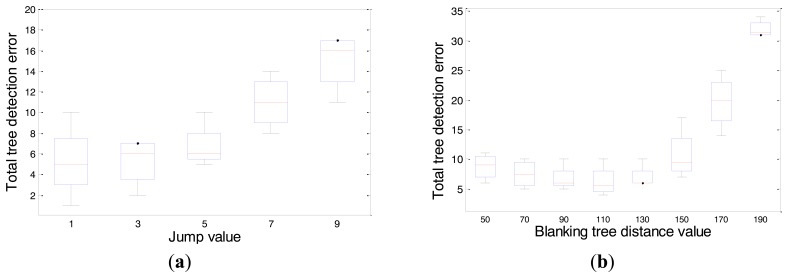
The total number of detected tree errors (false positives and negatives) by the LIDAR sensor using different parameter values: (**a**) Jump parameter with a Fischer value of 5.79. (**b**) Blanking tree distance parameter with a Fisher value of 24.44. On each box, the central mark is the median, the edges of the box are the 25th and 75th percentiles, and the whiskers extend to the most extreme data points.

Based on an independent set of evaluations (Figure 4), the values at which a minimum number of tree detection errors were obtained were as follow: a height cut of 21, an encoder range of 200, a path increment of 5 a cut identification of 19, a jump of 1, and a blanking tree distance of 110.

#### 2.3.3. Calibration Test II: Tree Classification

The trees that ware correctly detected by the LIDAR sensor in the 4 field tests comprised, 359 trees (284 alive and 75 dead), and they were used to calibrate the tree classification methodology. The methodology used for LIDAR tree classification was based on the following:

1. Considering the LIDAR detections that were inside the tree line defined in point 4 of the *“Calibration test I: Stem identification and selection of parameter values”* a binary transformation was performed to determine the presence or absence of detections for each encoder value.
2. Using the medium encoder values of each success tree detected in point 8 of *“Calibration test I: Stem identification and selection of parameter values”* as the midpoint in the binary transformations from the previous point, the number of presence detections was counted inside an evaluation window *(dead range parameter)*.
3. According to the number of detections inside the dead range window and the actual state of the tree, which was obtained manually during the test, the detection threshold *(threshold count parameter)* was obtained, which difference live trees from dead trees at 95th percentile of alive trees.

**Figure 5.**
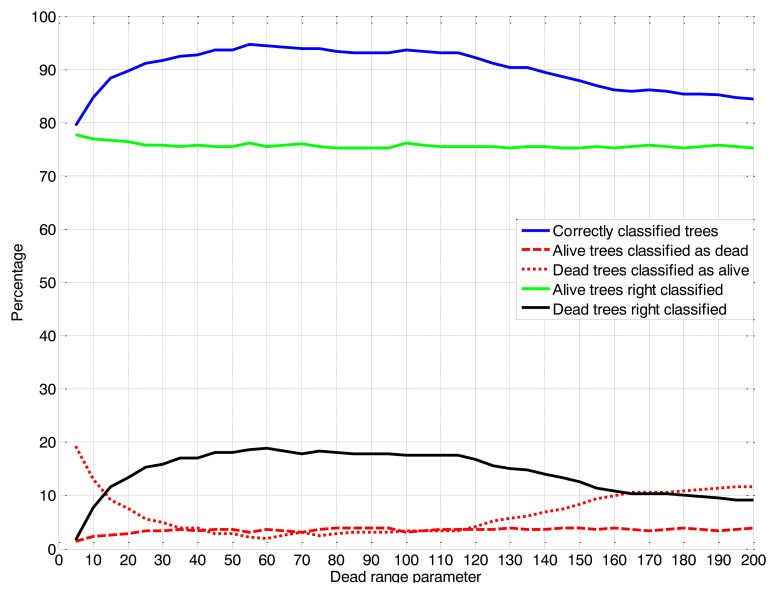
Classification tree percentages obtained for the different dead range parameter values by the LIDAR sensor.

This methodology was conducted through an evaluation window of the dead range parameter from 12.7 to 508 cm steps of 5 encoder values. Based upon on the validation tables (assessment of success and false positives and negatives), a dead range parameter value of 55 was selected (Figure 5). The success of the classifications (dead and alive trees) was considered along with the percentage of live trees classified as dead. For a nursery, this error should be as low as possible because it could involve the replacement of live trees and causes unnecessary expense to the nursery.

Once the dead range parameter was selected, the threshold count parameter was calculated in which the detection of live from dead trees was evaluated based on the 95 percent *(95th percentile*) of lives trees. Table 3 shows the mean validation values obtained in the calibration tests by selecting the highest, average and lowest threshold count value evaluated. To obtain a universal methodology, a single threshold count value should be calculated. To minimize unnecessary expenses to the nursery, the lowest threshold value (11.75 detections) was selected. This selection process involved a reduction of the error by which a living tree is considered as dead and the error whereby a large number of dead trees were considered alive.

### 2.4. Method for Light Curtain Tree Stem Characterization

Figure 1 shows the four pairs of light curtain sensors (model Mini-Beam SM31 EL/RL, Banner Engineering Co., Minneapolis, MN, USA) that were placed under the LIDAR device. The four light curtain receivers were configured to output a TTL pulse when the infrared beam was blocked by the passage of a tree stem during travel on the prototype vehicle. All four of the light-beam signals were monitored simultaneously in real-time by a high-speed embedded control system. This sensing system allowed for the analysis of within-row tree placement.

Light can be blocked by various circumstances, such as tree leaves, weeds, and large soil clods; therefore, unwanted pulses can be observed and cause inaccurate tree counting and sizing.

#### 2.4.1. Calibration Test I: Stem Identification

The data used for the light curtain calibration were the same as for the LIDAR calibration: 4 field experiments tests composed of 373 trees. The methodology used for the tree stem detection by the light curtain sensors was based on the following:

1. The detections of the three lower light curtain sensors (the highest sensor did not detect small nor dead trees) in an encoder window *“tree encoder parameter”* were evaluated using the successful detection of the previous/lower light curtain sensor as the midpoint. The evaluation order was upward, starting from the light curtain sensor located closest to the ground (LC0). For example, when detection at LC0 occurred at an odometer encoder value of 500 and with a tree encoder parameter of 100, the program assessed whether there was any detection in LC1 (light curtain above LC0) within a window range from 400 to 600 encoder values. If detection was obtained for LC1 in this range, the program evaluated the LC2 in the range of +/− 100 of the encoder value that produced the detection in LC1.
2. The detection was considered a tree if the condition of detection were fulfilled in LC0-LC2 (relative to the tree encoder parameter) and provided that the difference in the encoder values of this new candidate (LC0 encoder value) and the previous candidate were higher than the *“minimal tree distance parameter”*. If this was not the first tree detected, the program determined whether the distance between the previous tree encoder values was higher than the minimal tree distance (*i.e.*, not a repetition of the previous tree).
3. The LC0 encoder value for each candidate tree was compared with the actual values obtained manually during the test. The real tree location and light curtain tree detection were compared by proximity and deemed a success if the distance between them was less than 80 encoder values; otherwise, it was considered to be a false positive (detected by the light curtain but not real) or negative (real tree not detected by the light curtain).

**Figure 6.**
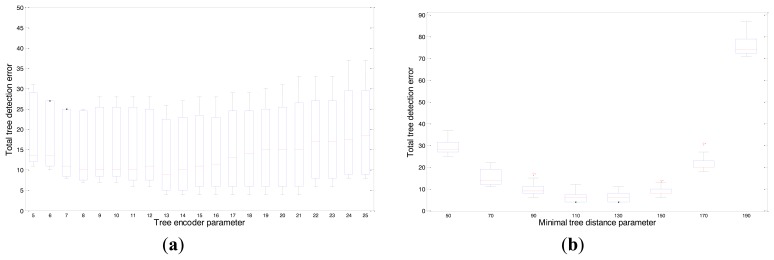
The total number of detected tree errors (false positive and negatives) by the light curtain using the different parameters values: (**a**) Tree encoder parameter with a Fisher value of 1.34. (**b**) Minimal tree distance parameter with a Fisher value of 621.7. On each box, the central mark is the median, the edges of the box are the 25th and 75th percentiles, and the whiskers extend to the most extreme data points.

This methodology was conducted through an evaluation range of the *tree encoder parameter* from 5 to 63.5 cm steps of 1 encoder values and a *minimal tree distance parameter*, from 127 to 482.6 cm steps of 20 encoder values. Table 4 shows the values of the parameters for which optimum results were obtained. Values were selected according to the total number of false detections (Figure 6). At equal false detections values, the standard deviation error was used as a selection criterion. Finally, a value of 13 was selected for the tree encoder parameter and 130 was selected for the minimal tree distance parameter.

**Figure 7.**
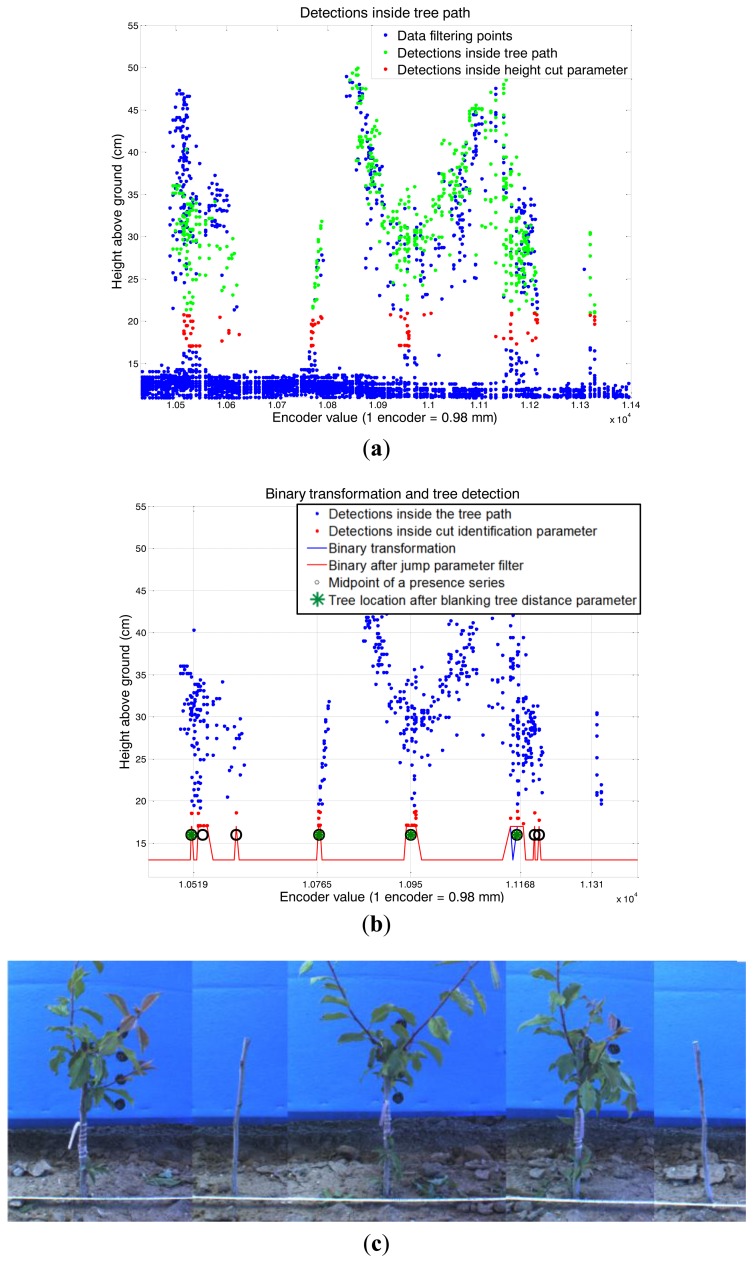
Methodology used for tree stem identification from the LIDAR detection data. In the graph on the right, the x-axis grid shows the actual location of the trees (the last one was not detected). The image shows the corresponding view of the scanned trees.

**Figure 8.**
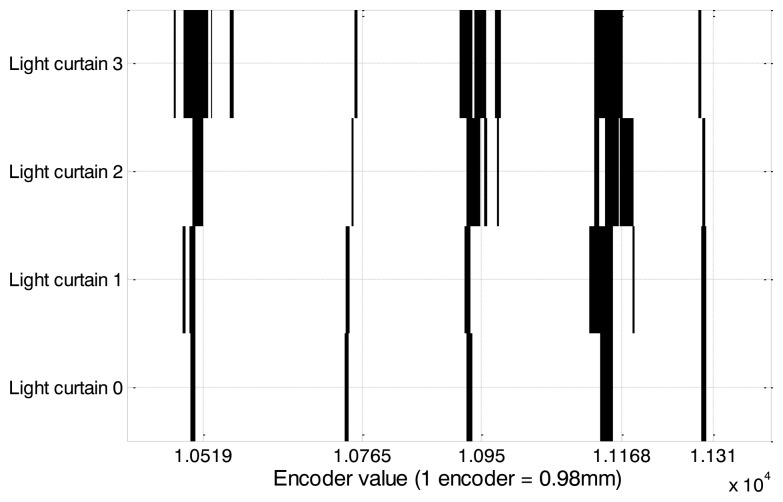
The x-axis grid shows the location of the trees detected with the light curtain sensor, and the y-axis shows which the light curtain sensor that is blocked by the tree in each location.

Figure 8 shows the same tree sequence as in Figure 7 but detected with the light curtain sensors. In this image, the light curtain sensor number 3 is blocked more frequently than 0, 1 and 2, which indicates that this sensor is frequently detecting leaves at the top of the trees.

#### 2.4.2. Calibration Test II: Tree Classification

To calibrate the tree classification methodology by the light curtain sensor, the number of successful detections of trees in the 4 field tests (371 trees with 294 alive and 77 dead) was used. The methodology was based on the methodology used for tree classification by the LIDAR sensor.

1. Considering the LC0 encoder value of each success tree detected (point 3 of *“Calibration test I: Stem identification and selection of parameter values”)* as midpoint, the total numbers of presence detections for LC0 to LC2 were counted inside an evaluation window *(dead range parameter)*.
2. According to the number of detections inside the dead range window and the actual state of the tree, which was obtained manually during the test, it was obtained the detection threshold *(threshold count parameter)*, which difference live trees from dead trees at *95th percentile* of alive trees.

**Figure 9.**
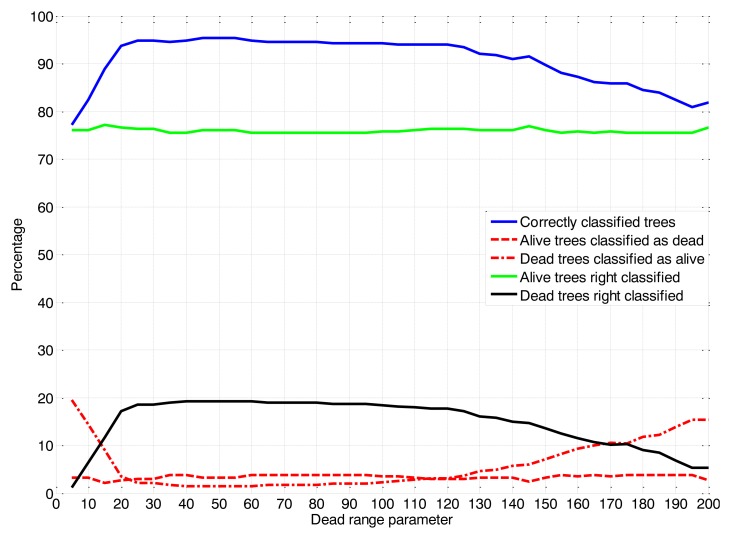
Classification percentages obtained for the different dead range parameter values for the light curtain sensor.

Similar to that of the LIDAR sensor, the evaluation range of the dead range parameter was from 12.7 to 508 cm steps of 5 encoder values. Considering the validation tables obtained for each dead range value (Figure 9) and the tree classification established by the LIDAR sensor, a parameter value of 50 was selected.

Once the dead range parameter was selected, the threshold count parameter was calculated in which the detection of live from dead trees was based on 95 percent *(95th percentile*) of lives trees. Table 5 shows the mean validation values obtained in the calibration tests by selecting the highest, average and lowest threshold count value evaluated. To obtain a universal methodology and minimizing unnecessary expenses to the nursery, the lowest threshold value (21.4 detections) was selected.

## 3. Results and Discussion

### 3.1. LIDAR Stem Identification Validation Tests

For the validation tests, two new field experiments were developed, composed of 194 trees along with the data used for the calibration. The methodology used in the calibration included the parameter values selected after the independent evaluations. The values obtained for both tests are summarized in Table 6, which shows that high percentages (95.7%) of trees were successfully detected, with a low location deviation error (total std. deviation of ±16.6 mm and a standard error of ±0.71 mm).

Tables 7 and 8 show the percentages and numbers of registered encoder values corresponding to each of the four possible situations during the LIDAR calibration and validation tests, which were the predicted and observed trees, predicted but not observed trees (false positive), observed but not predicted trees (false negative), and neither predicted nor observed trees. The percentages of the different situations obtained during the calibration and validation were very similar. Small variations may have occurred as a result of the different speeds used during the tests, and with each speed influencing the number of encoder values registered.

**Figure 10.**
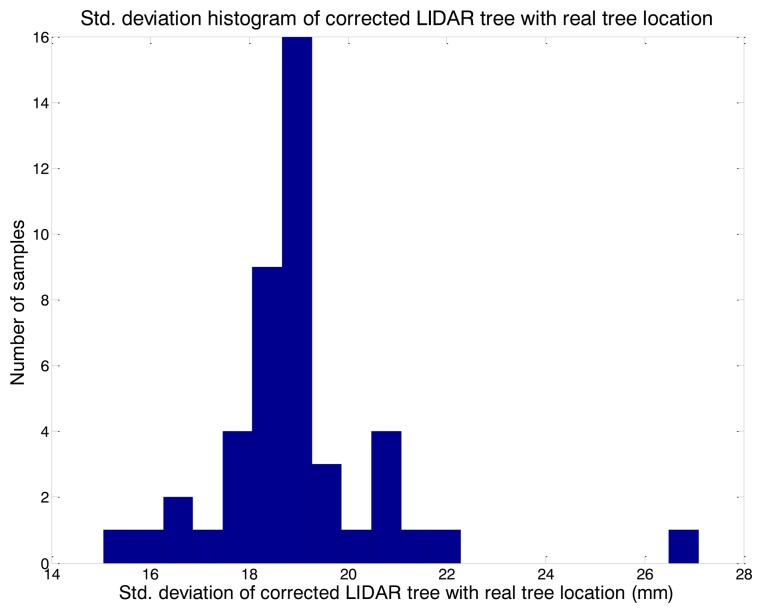
Histogram of the standard deviation error from the LIDAR tree location and real tree location.

Figure 10 is a histogram of the standard deviation of a correct tree LIDAR location and a real tree location. All of the tests were considered and grouped according to the parameter values during the independent evaluations. A low error value did not indicate an improved performance of the sensor because this may lead to a greater number of false positive or false negative tree detection. For example, using the lowest tree location error, which was 1 mm less than the actual observation, would result in a reduction in tree detection of up to 86%.

### 3.2. LIDAR Tree Classification Validation Test

Two new field experiments were developed that were composed of 184 trees (106 alive and 78 dead) along with the data used for calibration. The methodology used in the calibration included the parameter dead range at 55 encoder values and 11.75 counts for threshold. The values obtained for both tests are summarized in Table 9, which shows that 95.9% of live trees and 88.24% of dead trees were successfully classified, with 4.1% of live trees considered as dead and 11.76% of dead trees considered as alive.

Table 10 shows the mean validation percentage obtained for each of the four possible situations during the LIDAR calibration and validation tests, which were the predicted and observed live trees, predicted but not observed live tree (false positive), observed but not predicted alive trees (false negative), and predicted and observed dead trees.

### 3.3. Light Curtain Stem Identification Validation Tests

Table 11 summarized the data obtained using a value of 13 for tree encoder parameter and of 130 for the minimal tree distance parameter in all of the tests, which showed that 99.48% of the trees were detected successfully. Of the 194 trees that were in the validation study, 193 trees were detected after correction.

Tables 12 and 13 show the percentages and numbers of registered encoder values corresponding to each of the four possible situations during calibration and validation tests, which were the predicted and observed trees, predicted but not observed trees (false positive), observed but not predicted trees (false negative), and neither predicted nor observed trees. The percentages of the different situations obtained during the calibration and validation were very similar. Small variations may have occurred as a result on the different speeds employed during the tests influencing the number of encoders registered.

### 3.4. Light Curtain Tree Classification Validation Tests

The methodology used during the calibration was followed by setting the dead range at 50 encoder values and 21.4 counts for the threshold. The values obtained for both tests are summarized in Table 14, which shows that 97.28% of live trees and 86.16% of the dead trees were successfully classified, with 2.72% of the live trees considered as dead and 13.84% of the dead trees considered as alive.

Table 15 shows the mean validation percentage obtained for each of the four possible situations during the LC calibration and validation tests.

## 4. Conclusions

This study showed that the LIDAR and light curtain sensors represent a useful technique for within-row tree detection in a nursery. This study also developed an automated analysis for this type of technology that allows for the elimination of outliers, detection of weeds, tree leaves and soil based on point clouds detected by the LIDAR and light curtain sensors. Our major contributions are as follows:

- A sensor platform was successfully constructed to monitor and record the LIDAR and light curtain measurements simultaneously for a tree row.
- High percentages (95.7%) of trees were detected successfully with the LIDAR sensor, which also had a low location deviation error (total std. deviation of ±16.6 mm and a standard error of ±0.71 mm).
- Higher percentages (99.48%) of trees were detected successfully with the light curtain sensor, with a lower location deviation error (total std. deviation of ±11.32 mm and a standard error of ±0.48 mm).
- The LIDAR sensor correctly classified 93.75% of the trees compared to 94.16% for the light curtain sensor.

For the task proposed in this study, the most reliable system was the light curtain sensor. Not only were the best results obtained with this sensor, but also the data processing was much simpler, consisting of two filter parameters, rather than the six filter parameters required for the laser sensor. Further, reducing system complexity provides faster data processing, which is a plus for future applications in real-time.

From an economic point of view, the light curtain sensor, even though formed by four pairs of sensors, was less costly than the single laser sensor at a cost ratio of 2/1. The system could be used to automate intra-row (*i.e.*, within-row) weeding based on tree or crop detection with active optical sensors. In most cases, weed control still requires costly hand weeding for organic, nursery field and small-scale farmers.

The use of this innovative sensor platform for tree detection in nurseries may result in a new era that allows for online control of aggressive weeds and the automation of weeding tools, which we plan to pursue through future research. Further work is also required to provide additional insight into large commercial fields with different types of trees so that data obtained with the optical sensor can be related to the plethora of published studies that have used machine optical sensing.

## Figures and Tables

**Table 1.** LMS 221 technical data.

**Features**	**Performance**	
Operating range: Up to 80 m		Systematic error: ±15 mm
Angular resolution: 1°		Statistical error: ±5 mm
Light source: Infrared (905 nm)		Interfaces/mechanics/electronics
Field of view/Scanning angle: 180°		Data interface: RS 232 (38.4 kBd)
MTBF: 50,000 h		Supply voltage: 24 V DC (20 W)
Laser Class: 1 (EN/IEC 60825-1)		Enclosure Rating: IP 67
Scanning Frequency (by the Software): 10 Hz		Temperature Range: −30 °C to +50 °C

**Table 2.** Mini-Beam SM31 EL/RL technical data.

**Features**	**Interfaces/Mechanics/Electronics**
Range: 30 m	Output type: Bipolar NPN/PNP
Light source: Infrared (880 nm)	Supply voltage: 12 V DC
Maximal frequency: 500 Hz	Environmental rating: IEC IP 67
Beam pattern distance: ≈35 mm	Operating temperature: −20 °C to +70 °C

**Table 3.** Mean percentages of successful classifications and false positives and negatives during the calibration test with a dead range value of 55 at the different threshold count values.

	**Threshold Count Value**	**Alive Trees Correctly Classified (%)**	**Dead TREES Correctly Classified (%)**	**Alive Trees Classified as Dead (%)**	**Dead Trees Classified as Alive (%)**
Max value	18	69.29	19.18	9.86	1.67
Min value	11.75	78.06	16.93	1.09	3.92
Average value	15.75	75.60	18.06	3.55	2.79

**Table 4.** Results obtained during the parameter evaluation with minimal number of trees not detected (4 total trees not detected for a total of 373 trees).

**Tree Encoder Value**	**Minimal Tree Distance Value**	**σ of Location by LC with Real Values**
13	110	10.82
13	130	10.59
14	110	10.88
14	130	10.66
15	110	10.96
15	130	10.74
16	110	11.01
16	130	10.79
17	110	11.07
17	130	10.82
18	110	11.10
18	130	10.85
19	110	11.43
19	130	11.18
20	110	11.79
20	130	11.55
21	110	11.88
21	130	11.65

**Table 5.** Mean percentages of successes classifications and false positives and negatives during the calibration test with a dead range value of 50 at different threshold count values.

	**Threshold Count Value**	**Alive Trees Correctly Classified (%)**	**Dead Trees Correctly Classified (%)**	**Alive Trees Classified as Dead (%)**	**Dead Trees Classified as Alive (%)**
Min value	21.4	77.15	18.49	2.14	2.22
Max value	26	72.55	19.58	6.75	1.12
Average value	23.56	74.99	19.03	4.30	1.68

**Table 6.** Results obtained for stem identification with the calibration and validation samples using the parameter values selected from independent evaluations (height cut of 21, encoder range of 200, path increment of 5, cut identification of 19; jump of 1, and blanking tree distance of 110).

	**Calibration**				**Validation**		**Total**
Test number	1	2	3	4	5	6	
Real trees number	95	93	89	96	97	97	567
LIDAR tree counts	96	94	90	95	97	96	568
LIDAR tree correctly detected	91	89	87	92	93	91	543
False positives	5	5	3	3	4	5	25
False negatives	4	4	2	4	4	6	24
Total incorrect detections	9	9	5	7	8	11	49
σ of location by LIDAR with real tree values (mm)	17.79	19.26	17.94	12.25	16.38	11.86	16.6

**Table 7.** Number and percentage of encoder events recorded in each situation during the LIDAR calibration tests.

	**Predicted Trees**	**Unpredicted Trees**
Observed trees	1605 (7.4%)	140 (0.65%)
Unobserved trees	39 (0.18%)	19900 (91.77%)

**Table 8.** Number and percentage of encoder events recorded in each situation during the LIDAR validation tests.

	**Predicted Trees**	**Unpredicted Trees**
Observed trees	548 (7.28%)	100 (1.33%)
Unobserved trees	26 (0.35%)	6845 (91.04%)

**Table 9.** LIDAR results obtained for the tree classification with the calibration and validation samples using the parameter values selected in the calibration (dead range of 55 and threshold count of 11.75).

	**Calibration**				**Validation**		**Total**
Test number	1	2	3	4	5	6	
Real tree count	95	93	89	96	97	97	567
Trees detected by LIDAR after correction	91	89	87	92	93	91	543
Alive trees not detected by LIDAR	2	4	2	4	1	4	17
Dead trees not detected by LIDAR	2	0	0	0	3	2	7
Number of alive trees	76	75	68	65	54	52	390
Number of dead trees	15	14	19	27	39	39	153
Live trees well classified	75	75	68	62	52	42	374
Dead trees well classified	13	7	16	25	35	39	135
Alive trees classified as dead	1	0	0	3	2	10	16
Dead trees classified as alive	2	7	3	2	4	0	18

**Table 10.** Mean validation percentage using the parameters values selected for the LIDAR calibration and validation tests.

	**Predicted Alive Trees**	**Predicted Dead Trees**
Observed alive trees	69.05%	2.92%
Observed dead trees	3.33%	24.70%

**Table 11.** Light curtain results obtained for the calibration and validation samples using the parameter values selected after independent evaluations (tree encoder of 13 and minimal tree distance of 130).

	**Calibration**				**Validation**		**Total**
Test number	1	2	3	4	5	6	
Real trees number	95	93	89	96	97	97	567
LC trees counts	95	93	89	96	97	97	567
LC trees correctly detected	95	92	88	96	97	96	564
False positives	0	1	1	0	0	1	3
False negatives	0	1	1	0	0	1	3
σ of location by LC with real tree values (encoder)	8.8	13.7	9.13	10.74	13.21	9.35	11.32

**Table 12.** Number and percentage of encoder events recorded in each situation during the light curtain calibration tests.

	**Predicted Trees**	**Unpredicted Trees**
Observed trees	3140 (3.99%)	20 (0.02%)
Unobserved trees	2 (0.02%)	75460 (95.97%)

**Table 13.** Number and percentage of encoder events recorded in each situation during the light curtain validation tests.

	**Predicted Trees**	**Unpredicted Trees**
Observed trees	1257 (3.15%)	10 (0.03%)
Unobserved trees	1 (0.00%)	38637 (96.82%)

**Table 14.** Light curtain results obtained for the tree classification with the calibration and validation samples using the parameter values selected the in calibration (dead range of 50 and threshold count of 21.4).

	**Calibration**				**Validation**		**Total**
Test number	1	2	3	4	5	6	
Real tree count	95	93	89	96	97	97	567
Trees detected by LC after correction	95	92	88	96	97	96	564
Lives trees not detected by LC	0	1	1	0	0	0	2
Dead trees not detected by LC	0	0	0	0	0	1	1
Number of alive trees	78	78	69	69	55	56	405
Number of dead trees	17	14	19	27	42	40	159
Live trees correctly Classified	74	78	67	67	54	54	394
Dead trees correctly classified	17	10	15	27	33	35	137
Alive trees classified as dead	4	0	2	2	1	2	11
Dead trees classified as alive	0	4	4	0	9	5	22

**Table 15.** Mean validation percentage using the parameters values selected for the light curtain calibration and validation tests.

	**Predicted Alive Tree**	**Predicted Dead Tree**
Observed alive tree	70.09%	1.94%
Observed dead tree	3.90%	24.07%
